# Nursing staff interactions during the older residents’ transition into long-term care facility in a nursing home in rural Norway: an ethnographic study

**DOI:** 10.1186/s12913-015-0818-z

**Published:** 2015-03-29

**Authors:** Marianne Eika, Bjørg Dale, Geir Arild Espnes, Sigrun Hvalvik

**Affiliations:** Department of Social Work and Health Science, Faculty of Social Sciences and Technology Management, Norwegian University of Science and Technology (NTNU), 7491 Trondheim, Norway; Telemark University College, Faculty of Health and Social Studies, Post box 203, , NO-3901 Porsgrunn, Norway; Center for Caring Research, Telemark University College, Post box 203, , 3901 Porsgrunn, Norway; Department of Health and Nursing Sciences, Agder University, Campus Grimstad, Post box 509, , N-4898 Grimstad, Norway; Center for Caring Research – Southern Norway, Campus Grimstad, Post box 509, , N-4898 Grimstad, Norway

**Keywords:** Long-term care facility, Staff interactions, Transition, Complexity science, Resident, Ethnography

## Abstract

**Background:**

Future challenges in many countries are the recruitment of competent staff in long-term care facilities, and the use of unlicensed staff. Our study describes and explores staff interactions in a long-term care facility, which may facilitate or impede healthy transition processes for older residents in transition.

**Methods:**

An ethnographic study based on fieldwork following ten older residents admission day and their initial week in the long-term care facility, seventeen individual semi-structured interviews with different nursing staff categories and the leader of the institution, and reading of relevant documents.

**Results:**

The interaction among all staff categories influenced the new residents’ transition processes in various ways. We identified three main themes: The significance of formal and informal organization; interpersonal relationships and cultures of care; and professional hierarchy and different scopes of practice.

**Conclusions:**

The continuous and spontaneous staff collaborations were key activities in supporting quality care in the transition period. These interactions maintained the inclusion of all staff present, staff flexibility, information flow to some extent, and cognitive diversity, and the new resident’s emerging needs appeared met. Organizational structures, staff’s formal position, and informal staff alliances were complex and sometimes appeared contradictory. Not all the staff were necessarily included, and the new residents’ needs not always noticed and dealt with. Paying attention to the playing out of power in staff interactions appears vital to secure a healthy transition process for the older residents.

## Background

In developed nations, there is an expected increase in the number of older people above the age of 67 [1]. In Norway the number of people above the age of 80 is estimated to double over the next 35 years. Due to the increasing number of older frail people and a decrease in the number of people to take care of them, there is a growing concern for the future recruitment of competent nursing staff to nursing homes [2,3]. Older people in long-term care facilities (LTCFs) have complex medical and care conditions [4,5] and require competent care. Internationally, in contemporary health care environments for the elderly, the employment of unlicensed staff in direct patient care is on the increase [6,7]. Researchers [6] have noted that there has been a paradigmatic shift in staffing outcome literature from “an individual to team mindset” (p 10), emphasizing teamwork and inter-professional collaboration. Harris and McGillis [6], conclude that administrators and researchers need to pay attention not only to skill mix and numbers of staff, but also to processes of interaction between patients, providers and organizations.

Staff interaction plays a role in the quality of care to older people in nursing homes [8-10], and the challenges are to use the available personnel in the best possible ways to promote good quality care. There are no national guidelines for formal staffing levels in nursing homes in Norway [11]. Yet, the licensed staffing levels are relatively high, compared to other European countries [12]. According to a study of 12 nursing homes in 4 of the largest municipalities in Norway [13], registered nurses constituted 24,1% of the workforce, auxiliaries 46,3%, and unlicensed assistants 29,6% on weekdays. During weekends the unlicensed assistants constituted 47,6% of the workforce. Varieties of primary nursing systems are used in many Norwegian nursing homes. The primary nursing care delivery model [14], support a patient-centered nurse-patient relationship that promotes continuity of care. Each patient is assigned one primary nurse who assumes responsibility and authority to “assess, plan, organize, implement, coordinate, and evaluate care in collaboration with the patients and their families” (p 295).

Moving into LTCF is a stressful change [15-17] for older residents and their family members, and can be associated with the concept transition defined as a passage between two relatively stable periods of time where the person moves from one life phase, condition or status to another [17]. It refers to processes and outcomes of complex person-environment interactions [18]. A transition may be triggered by a change or marker event, which may bring a period of upheaval and disequilibrium for the person (s) involved. Nursing research show [17] that transitions may connect with uncertainty, emotional distress, interpersonal distress and worry. Needs may not be met in familiar ways and changes in the person’s self-perception and self-esteem are common. Transition theory in nursing is useful in the development of nursing therapeutics to facilitate the transition that people undergo. According to Meleis et al. [18], nursing therapeutics involves nursing strategies during transition enabling the nurses to anticipate points at which the person is most likely to reach peak of vulnerability, and select “the most fruitful kinds of action and optimal intervention points for achieving the desired health maintenance or health promotion goals” (p 29). Possible interventions include continuous assessment, reminiscence, role supplementation, creation of a healthy environment, and mobilization of resources [17]. Geary & Schumacher [19] suggest integrating concepts of complex adaptive systems from complexity science to connect the transitions to the context in which they are occurring. Transition is a process, not a change that occurs in a moment of time [18,19], and complexity science [19], “illuminates the nature of the transition process and changes that occur while a transition is unfolding” (p 241). During interactions, new processes or patterns of interaction, as well as new outcomes, emerge. Concepts suggested [19] are multiple individual agents interacting locally in a dynamic non-linear fashion, relationships, self- organization, emergence, and culture and environment of all agents. Self-organization refers to new behaviors or new patterns that emerge from individual agents’ reaction to changes within the complex organization. Although self-organization “appears to be planned within the system, it is actually the reaction of an agent or a group of agents to change made by another. With the new information, agents, acting on established rules, change their behavior, leading to a new structure” ([19], p 239). Emergence refers to changes that are not predictable for two reasons, the absence of a complete context, and that interactions between persons are nonlinear.

This paper is part of a larger study exploring and describing the transition of older people into LTCF in a nursing home in southern rural Norway from the perspective of next-of kin and staff. In our first study [16], we explored the experiences of next-of kin during their older family members’ transition into LTC-placement. In the second study [20], we explored and described different nursing staff’s actions during the older residents’ initial transition period in the LTCF. This present study focuses on staff interaction based on the same ethnographic data as in the second study. Literature exploring staff interaction within residential long- term care for the elderly has different perspectives and foci. One study explores the challenges between licensed and unlicensed staff working together [21], others explore and support the empowerment of direct care workers [8,22], and yet others explore and describe interaction patterns among all nursing staff [10]. We have been unable to find studies dealing with licensed and unlicensed staff interactions during older residents’ transition into LTCF.

The aims of this study were to explore and describe the nursing staff interactions during the older residents’ transition into LTCF, and how staff interactions may influence their assistance and care for the older residents in transition.

## Methods

An ethnographic design helped gain in-depth understanding of staff interaction in contexts [23,24]. Humans are social beings whose actions, opinions and self-understanding are influenced by context, and influence back on context [25]. The ontological position taken was constructivist [26] with an analytical middle ground between reality and representation. In the hermeneutical tradition of Gadamer [27], the concepts horizon and prejudices closely link with the identification of the researchers’ pre-understandings as part of exceeding one’s horizon. It posed challenges that the authors have a background as registered nurses with an interest in the care of older people. While our preconceptions and knowledge could give an easier understanding of what was going on, we could also become too familiar and understand too quickly. Daily critical reflection during the participant observation periods and interviews helped to use our preconceptions in critical and constructive ways [27,28].

Rigor was established by the time frame of the study, and by using multiple methods in data collection. The analysis was undertaken in collaboration with an experienced researcher in qualitative methods.

### Data collection, context and participants

Three sources of data were used; periodic participant observations, interviews and reading of documents. Periodic participant observation periods following ten new residents on admission day and the initial week were performed, commencing early June 2011 and ending January 2012. The head nurse contacted the researcher about the expected arrival of a new resident. The researcher was present in the LTCF during the preparation period before the resident arrived, on admission day and the first week. The selection of staff participants were mainly those who were appointed primary contacts for the new resident, and those they interacted with. The participant observations were carried out to get insights into staff’s interactions in different settings such as meal situations, new residents’ morning care and oral shift reports, to name a few. The researcher was in the facility during daytime and afternoon shifts weekdays, weekends and summer holiday. Writing memos was carried out as soon as possible after something had taken place. When the researcher did not directly participate, for instance during a meal or a shift report, notes were taken as the event evolved. This strategy was used when the staff had become familiar with the researcher’s presence. Further, the researcher’s reactions and reflections were written down daily to identify prejudices and role confusions brought to the study. These intermittent periods posed challenges both for the researcher’s own role understanding, and in ensuring that all the staff involved in the study at any given time were informed about the researcher’s role, and the purpose of the research project. The sporadic participant observation periods opened up to perform some of the semi-structured interviews in-between. This combination helped clarify issues that were unclear, and directed the subsequent observation periods and semi-structured interviews. The written material such as the individual plan on the computer, the care plans in the residents’ bathrooms, and daily written reports were consulted [29], mainly to confirm and augment data from other sources. Individual semi-structured interviews with seventeen staff members comprising four nurses, six auxiliaries, five assistants, the head nurse, and the leader of the institution were carried out in a small room in the nursing home outside of the LTCF. The recruitment of the respondents to the formal interviews were by voluntary participation, and some were headhunted by the researcher as the study went on. Each interview lasted an hour on average. The interview guide had questions about how staff interacted with colleagues during the preparation period, admission day and initial week after arrival. The first author attempted to follow what the respondents themselves associated and found relevant to talk about relating to this topic. The interviews were audio recorded and transcribed as soon as possible after they had taken place. The weekly periodic participant observations opened up for the researcher to have informal conversations with the new residents, and this influenced further fieldwork and the analysis.

The nursing home is situated in rural southern Norway. The LTCF consists of thirty private rooms split into three units each with ten private rooms. The nursing staff comprises licensed registered nurses (nurses), auxiliaries (auxiliaries), and unlicensed care assistants (assistants). The nurses have three years’ nursing education from university/university-college and the auxiliaries have two years’ training in high school [30]. The unlicensed assistants were not educated in health care apart from short courses at the workplace. The participants were females with two exceptions. The age ranged from early 20s to early 60s, and the length of employment varied from a few weeks to more than 30 years. Staffing ratios and mix varied with the shifts, weekdays or weekends, and holidays. During daytime on weekdays, the staffing was three staff to ten residents, and usually there was one nurse to ten residents or sometimes one nurse to five residents. The auxiliaries or assistants ratio was one staff to three or four residents. In the evenings, there were usually two staff (auxiliaries or assistants, or both) in each unit and one nurse in charge of the facility. In addition to permanent licensed and unlicensed staff, there were part-time supply assistants who worked weekends only. All staff categories performed direct resident care. The care was organized according to a primary nursing model [14], meaning that in this LTCF the nurses were responsible for five residents each, and the auxiliaries normally shared responsibility with the nurse in each unit for three residents each.

### Data analysis

The first author immersed herself in the transcribed interviews and field notes as the study went on and after the collection of data was over. Writing is a key part of the entire research process, and closely related to analysis [23].Writing things down during the fieldwork and interview periods, and then writing things up [24] helped in this endeavor. The interview texts and the fieldwork texts were treated as texts equally important [31]. Sensitizing concepts suggested further directions in which to look, and gave us “a general sense of reference and guidelines in approaching empirical instances ([23], p 164, based on Blumer [32]). Some concepts were “the physician’s round”, “primary nursing”, “open door” and “chameleon.” The written documents were consulted mainly to check our understanding of the data from the interviews and the fieldwork [29]. The data were read repeatedly and in different ways to get different versions [23,33]. The parts of the transcribed interviews and field notes dealing with the same issues were taken out of the contexts in which they occurred, to help get hold of the different versions of the same phenomenon [33]. For instance, “spontaneous staff interactions” was connected across the data material. The researchers also searched for theoretical perspectives that helped make sense of the emerging patterns [23,24] central to the aims. We then checked if what we had interpreted from the material taken out of context was in accordance with the contexts where they occurred [33]. If not, we started all over again. The emerging themes were further explored to clarify their meaning and explore their relation to other themes. The sub-themes show the variations contained in each theme.

### Ethical considerations

The Regional Committees for Medical and Health Research Ethics in southern Norway approved the project (REK 2011/153b). Formal access to the field was granted through the health care authorities in the municipality. Participants were assured confidentiality, informed that their participation was voluntary, and that they had the right to withdraw at any time without stating a reason. Written informed consent was obtained from all staff participating in the interviews, and all agreed to the interviews being recorded. A staff information meeting was arranged prior to the fieldwork. Residents were asked orally and in writing if they accepted that the first author participated in their daily care in the first week after arrival. Eight residents consented while two residents were considered cognitively impaired, and next-of kin consented on their behalf.

## Results

The analysis consists of three overall themes with several sub-themes, which illuminate how staff interacted during the older residents’ transition into LTCF, and possible influence on patient assistance. The following identified overall themes: The significance of formal and informal organization; interpersonal relationships and cultures of care; professional hierarchy and different scope of practice. The themes overlap and intertwine in complex ways.

### The significance of formal and informal organization

The staff interactions appeared modulated by the primary nursing model, the head nurse management style, and staff mix at different shifts. Their interactions were influenced by, and influenced back on, individual actions and team work, and information flow.

#### Individual actions and team work

Often the staff in the small units appeared to interact continuously while assisting and assessing the new residents. They acted in coordinated ways with their colleagues regardless of professional level while attempting to adapt to the new resident’s evolving needs. Yet the staff interactions were to a certain extent characterized by the understanding of their work as individual actions. Due to the primary nursing model, most licensed permanent staff were responsible for three to five residents each. They attempted to cater for most aspects of the new resident’s needs, and this ambition put pressure on each staff member. For instance, one primary nurse was at work on her day off to talk to family members after a newly arrived resident had died. In addition, a part-time primary nurse wanted to get an overview of all the residents in the facility, and thus worked extra. The nurses claimed that the primary nursing model in each unit meant that no nurse had an overview of all the residents in the facility. Furthermore, those who chose to work part-time, on average in eighty percent positions, did so to have the strength to do a proper job. If they considered their job well done, it gave them energy to accomplish the little extra for the new residents and the residents in general.

Connected with the notion of total responsibility for the new resident, the staff viewed their own work and that of each other differently. Many auxiliaries felt that they knew more about the new residents’ overall needs initially than the nurses, because the nurses had so many other tasks to perform in this period. It appeared in the interviews and the participant observation periods that most nurses regarded it as self-evident that they knew most about the new residents’ psychosocial as well as medical needs. They argued that even though they had many different tasks to perform initially, they still spent a lot of time with the resident in the small units. In addition to many fragmented tasks to perform concerning the new residents, the nurses had to prioritize those residents in most need. Sometimes this was at the expense of interacting and collaborating with staff colleagues during the shifts, and could restrict their face-to-face interaction with the new residents.

The primary contacts, the nurses and auxiliaries, had authority among the staff, and few colleagues wanted to interfere. In their absence, some were reluctant to perform independently towards the new resident, illustrated by the following:

When the primary nurse was on sick leave, another nurse was responsible for that unit. She did not establish documentation areas in the computer care plan, and argued that she would not interfere with the ways her absent nurse colleague worked. This made it difficult for the other staff to document in the computer program the first days after the resident’s arrival (fieldwork observations).

During evenings, week-ends and holidays, the mix of staff could disturb the primary nursing arrangement, illustrated by the following:

If only supply staff worked in one unit during a shift, the primary auxiliary could be transferred from her unit to compensate for the shortage of licensed personnel in the other unit. This was frustrating because she did not have the chance to follow up the newly arrived resident as well as she would have liked (summary of parts of interview with auxiliary).

These circumstances disturbed the permanent staff’s work rhythm with their primary residents. Some found supply staff a nuisance to work with mainly because of this.

In these periods with many part-time supply staff at work, the care appeared crudely performed. It seemed that the regular staff helped the new residents to settle in, while some of the new residents withdrew with many supply staff at a shift:

Even though many residents preferred to spend time in their rooms between meals, it was exceptionally quiet in the units at shifts with many supply staff at work. It seemed the cognitively able new residents quickly learned to take after the other residents’ strategies at these times; after the meals, they went into their rooms and shut the door behind them (fieldwork observations).

The head nurse (HN) attempted to support each staff’s self-confidence and self-reliance in their interaction with the residents, “to make them aware how much each one of them matters” (interview HN), and she kept her door open when she was in her office. It varied among the staff how they related to this. Some consulted her frequently, while others said that the HN was often away at meetings. Still others, like the week-end supply staff, never had the opportunity to interact with the HN in this way. This management style encouraged individual staff to develop a relationship with the new resident at their own speed. Furthermore, it seemed to legitimize that the staff nurses managed their units differently. This could cause problems particularly for the nurse in charge of a night or weekend shift, who had responsibility across all three units. For instance, written information on paper about the new resident was stored in different places in the three units, and the nurse in charge spent a long time before she found the papers.

#### Information flow

There was a dominant oral culture in the LTCF and its units, and face-to-face communication was the most common. Often the unit staff interacted spontaneousl*y* by sharing information and brainstorming together to help the new resident. Some staff could dominate in the oral culture irrespective of formal position, which frustrated some assistants:

As unlicensed staff it is very difficult – eh it often happens that you are trapped between two who have strong opinions about how to care for the new resident, right? Eh, sometimes one feels like a chameleon - that one goes into the roles of those one works with at any given time (interview assistant).

This assistant frequently consulted the HN when she was available, and these interactions contributed to strengthening her self- esteem and belief in her own skills.

Particularly the auxiliaries and assistants perceived that they had neither the time nor the calmness to read about the new resident in the computer program “while colleagues were toiling in the units” (interview auxiliary). Some were apprehensive that their colleagues could interpret sitting at the computer as avoiding work.

The assistants felt that they sometimes lacked information about the new resident, and how to perform their work. They had some initial training in the facility before they started, but had to tackle many things ad hoc. They generally wished the permanent staff to inform them better. “It is easy to forget to inform colleagues when one has been working for a while, and knows one’s way around” (interview assistant). Some assistants admitted that they should ask when in need, but were afraid of asking about something they believed everybody knew, and sometimes they did not know what to ask about. The potential consequences for the new residents were that everyday basic needs and observations were unnoticed, or if noticed, would not be passed on to colleagues. The assistants’ lack of knowledge about the new resident’s needs could be uncomfortable, for the new resident and the assistants, illustrated by the following:

It was during one of the first shifts I worked after some time off and I assisted a new resident whom I did not know. I just poured milk into his glass and gave it to him. He coughed a lot and I was afraid he would choke. I learned afterwards that he should have had “Thick and Easy”, instant food thickener, added to his milk to make it easier for him to swallow. Nobody told me and it was not written anywhere – so such things are easily forgotten and taken for granted that everybody knows ……..so poor resident, he coughed and hawked during the entire breakfast (interview assistant).

This assistant read about the new resident when she arrived back at work after some days off, and discovered that this important piece of information was unwritten. It appeared to be an attitude among many staff that it was little point in reading, which again seemed to encourage an attitude of writing less. Furthermore, the permanent licensed staff, particularly the night shift staff, could lack information about the new resident. At the oral shift reports staff did not have the time to report all aspects of the new resident’s condition and needs to the staff at the next shift, and the next shift staff did not always read the new resident’s individual protocol:

The night shift staff was unaware that the new resident was incontinent for feces, and did not look into his room during the night rounds. This information was not passed on at the oral shift report, but was written in the computer care plan. Since the new resident did not want to disturb the night staff, he tried to manage on his own. He made a mess and felt very bad about it. He had poor vision and it was difficult for him to tidy up after himself, and he needed help. Regarding this resident, the oral interactions among staff in the initial period did not focus on his physical shortcomings (fieldwork observations).

The taken-for-granted attitude among permanent staff combined with little or no writing or reading, made it even harder for the staff in need of information about the new resident. There were serious consequences for the new resident. Even though the staff could adjust to the new resident’s evolving needs, the care could also be based on general principles of care instead of tuning in to the new resident’s particular needs and preferences. For some new residents the unpredictability of the assistance was disheartening.

### Interpersonal relationships and cultures of caring

The staff interactions were influenced by, and influenced, intra- and inter -professional collaboration, personal traits and attitudes, and professional authority.

#### Alliances and collaboration

Staff collaboration appeared strong in intra- professional alliances. The general pattern was that individual staff appreciated working with people similar to themselves, and some met each other in their spare time. Typical for most alliances was a need of sparring with partners with the same values and outlooks of good patient care. Having a partner (s) helped the individual staff stick to their ideals and norms. Some felt they could accomplish more, and exploited the shifts they worked together to do it their way and accomplish little extras such as bringing strawberries for the afternoon coffee. The nurse alliances helped to strengthen their belief in their own professional judgments, and influenced their authority. Those who were not so strongly involved in alliances said that they felt insecure and inferior to some authoritative nurse colleagues.

The alliances seemed to influence the assistance of the new residents in different and unpredictable ways. Some allies focused on the new resident’s emerging needs and attempted to assist in their best interest, while others would rather “satisfy your relationships with colleagues than assist the residents (interview nurse).

The staff also collaborated inter-professionally, and the primary nursing arrangement in the small units encouraged such interactions. Yet it seemed to some extent to depend on individual staff and those working together at any given time. For instance, the collaboration between the assistants and the other staff appeared to depend on person. Some assistants seemed to have more authority than others, and be more part of the unit team. Mostly, the assistants kept in the background in staff interactions, and permanent staff appeared to make few efforts to include them in discussions about the new resident.

Regardless of alliances, most permanent staff missed regular formal meetings. The meetings were cancelled mainly because key persons such as the nurses were absent, or too busy. Some auxiliaries claimed these meetings would provide them with the same medical information from the nurses about the new residents, and make them more confident in their observations and assistance of the new residents. In addition, the auxiliaries appreciated being in a setting of dialogue and discussions, where everybody had the chance to participate.

#### The privacy of caring

Some auxiliary allies were strongly involved with the residents and provided extras such as making cookie dough at home for the residents to bake, bringing local poetry to read, and arranging parties. Full-time employees and nurses with their professional focus had neither the time nor the energy to be so involved in these activities. The head nurse attempted to even things out, so that everybody felt their work appreciated. Most new residents appeared at ease participating in familiar everyday activities. One new resident was provoked, however, when asked to participate in the baking of Christmas cookies the day after he had arrived. He claimed the activity was a fake. He had other needs at this time, such as getting help with his diahorrea, and come to terms with being in the LTSF (field observations).

### Professional hierarchy – different scopes of practice

The staff interactions were influenced by, and influenced back on, the professional hierarchy and the different staff’s perceived responsibility and work domains.

#### Hierarchy and responsibility

The staff awareness of the professional hierarchy varied. The assistants talked about “being at the bottom” while the auxiliaries expressed that “we are not so high up in the hierarchy”. The nurses did not explicitly talk about it, but appeared self-conscious about being the leaders.

Some assistants seemed comfortable with not having the same responsibility as the others. The danger was that they took for granted that the licensed staff knew what they knew, and would see to it. Some did not report obvious everyday observations about the new residents, which may be considered negligence on their part. One assistant said that she sometimes kept quiet when she knew something about the new resident that the permanent staff did not know, because she was “only an assistant” Illustrated by the following:

When two staff had to assist the new resident in the morning care, the assistant knows in detail how the new resident prefers his assistance because she has helped him in previous morning care situations. The licensed nurse/auxiliary, however, may assist the resident for the first time, and she is paying little attention to the resident’s preferences and abilities and the assistant’s knowledge and experience (summary part of interview assistant).

Moreover, the assistants believed that if the permanent staff regarded them as incompetent they might lose their job. The fact that many permanent staff appeared not to expect the assistants’ participation in discussions about the new resident could reinforce the mechanism of assistants being exempt from responsibility. One assistant felt personal responsible and would have appreciated information from the permanent licensed staff. For instance, she had to ask to get supervised in the Heimlich maneuver, which is a technique for preventing suffocation when a person’s airways become blocked. She had expected the permanent staff to inform her about that.

#### Monopoly of medical knowledge

It appeared as self-evident for the management and most nurses that the nurses and the physician had the monopoly of the body of medical knowledge. The physician expected the nurses to prepare his once-a-week round properly, so that he could perform his work efficiently. This could keep the nurses away from interaction with colleagues and the new resident in the initial transition period. The primary auxiliaries perceived this round as a “secret meeting” between the physician and the nurses:

We are not nurses and we are not physicians, and we know that, and I believe we do not trespass into their professional territories. I believe we are very conscious about that. Yet we are knowledgeable, but we are never asked. The physician never asks us about anything. If the staff nurse is absent, a nurse from another unit who does not know the residents joins the physician on his round. I think we could have done that, too. We auxiliaries are, however, not high up in the hierarchy. I like my job and I do my best and don’t care if I am not so high up there. I have collaborated with the occupational therapist and she listens to us and acts on our observations. We cooperate well and find the best solutions together” (interview auxiliary).

This quote illustrates an attitude among many auxiliaries that good collaboration was to “find the best solutions together” regardless of professional position. Moreover, many connected staff collaboration with knowing one’s limitations: “Residents trust us when they know that staff cooperate well and know their limitations” (interview auxiliary).

Many auxiliaries, assistants and supply staff would have appreciated that the nurses supervised them orally after the physician’s round. Sometimes this happened, but was not a pattern. The nurses seemed to have different opinions about the auxiliaries’ involvement in medical matters. Some stressed that “If one wants to help the patients in the best possible ways one has to involve everybody who is together with the patient” (interview nurse), and argued that it was unrealistic for the nurses to manage all the follow-up on their own. Not all the nurses or the management seemed to share this view.

When a new resident arrived, the nurse presented herself to the resident and his/her family as the sole primary nurse, and omitted mentioning the primary auxiliaries. If the next-of-kin asked for information about their older resident, the primary auxiliaries were frustrated when they had to direct them to a nurse who might not know the resident at all but knew the resident’s medical situation. The consequences for the new residents could be that critical observations and knowledge about their everyday needs, preferences and medical condition were ignored initially.

## Discussion

The aims of this study were to explore staff interactions, and how the interactions may influence the care of the older residents in transition into LTCF. The findings reveal complex staff interactions, and suggest that this influenced how they assisted and cared for the new resident.

The HN’s relationship-orientation seemed to encourage individual staff in their interaction with her, and the new residents. She appeared to create a climate that inspired possibilities and safety among the permanent staff. We wonder, however, if sometimes her involvement with individual staff could impede the team interactions. When the HN was available it seemed easier for individual staff to discuss their imminent concerns about the new residents with her. These interactions appeared to give individual staff authority among the other staff since they had consulted the head of the facility. According to studies [10,34,35], management support of good relationships among staff such as building connections and developing existing strengths, contribute to the delivery of better resident care and foster staff interdependence. The HN attempted to balance structures and routines with building individual staff’s self-confidence in spontaneous interactions with the new resident. This management ideology may connect with complex adaptive system’s theory. According to Penprase & Norris [36], this theory frees nurse leaders from a management that prescribe behaviors that stress prediction and control, to behaviors that aim to build strong relationships with the freedom to produce creative outcome. “Allowing teams to form on their own encourages a culture of care and connection in which staff are highly responsive to the needs of their units“(p 128). Transition theory in nursing [18], also underscores that the agents react to the emergent changes in flexible and dynamic ways. Still, the HN’s balanced approach appeared contradictory, at the same time as the HN provided feedback and praise to mainly the permanent staff, this individual staff focus could contribute to less focus on staff interactions that promoted good quality care. Research suggest [10] that “managers should scan their facility for existing pockets of excellence, to discover, support and expand staff interactions and relationships that already promote better performance” (p 13).

The arrangement with the primary nursing model influenced staff interactions in complex and at times contradictory ways. This way of organizing the work supported the dynamic, inter-professional staff collaborations among the primary contacts, where staff discussed their observations and uncertainties concerning the new resident. Still, each primary contact was assigned their role in the primary nursing teams. This organization appeared to legitimize that some did not fully involve themselves with each other and the new residents at shifts where the primary nurse and auxiliaries were absent. Needs considered unnecessary to deal with immediately, were left to the primaries to take care of when they were back at work. Research regarding the relationship between the primary nursing model and the quality of care is inconclusive [14]. Furthermore, the findings suggest that the primary nursing arrangement contributed to gluing the primaries to their individual responsibility beyond their paid work responsibilities.

The staff in the units also formed their own teams. Often, in these situations, everybody contributed regardless of professional competence, and noticed and assisted the new residents’ emergent needs. This can be associated to research pointing at physical infrastructure [37] as one necessary component for successful staff collaboration. Each unit was small and encouraged the staff to continuously interact and complement each other. While the nurses focused predominantly on the new residents’ medical condition, the enthusiasts provided good care in a homelike atmosphere. According to a study [38], quality care comprises attention to psychological and social needs along with medical considerations.

Moreover, Geary and Schumacher [19] argue that open boundaries between the agents “provide the potential for interactions that enable self-organizations, sense-making, and emergence of agent-specific processes and outcomes” (p 244). Our findings suggest that at some times, in some situations and depending on persons involved, boundaries were more open than at other times. It appeared that the spontaneous interactions per se contributed to creating open boundaries among the staff. Leykum et al. [39], found in their analysis of eight observational and interventional studies that how individuals self-organized was not necessarily done according to hierarchy or organizational structure but “based on how the work is actually accomplished” (p 2). In our study, the staff self- organization appeared based on structural features as well as how the work was carried out.

The arrival of a new resident in the LTCF and particular unit changed the work environment. Literature [40] has identified key practices that allow organizations to adapt successfully to such changes. One is to let information flow spontaneously among all agents. Our study focused on staff as agents, and although information at times flowed spontaneously among them, the findings also demonstrate otherwise. The lack of medical knowledge among the auxiliaries and assistants and sometimes the nurses’ lack of personal involvement and knowledge about the new residents’ everyday needs could contribute to fragmented understandings and resident assistance during this period.

Aspects of authority and power within the LTCF influenced staff interactions, and the findings disclose intricate power mechanisms at play. The oral culture allowed some to dominate, between staff groups as well as within staff groups. Eloquent persons got their opinions through, sometimes at the expense of sound professional knowledge and colleagues’ well-being. Being a member of an alliance gave some power and authority, sometimes at the expense of others, and staff collaboration on a more general level was disrupted. The unlicensed staff were often not fully involved in the staff collaborations and discussions about the new residents. Not being involved may have negative consequences in several ways. This is in accordance with studies [40,41] which show that well- informed and supervised nursing assistants perform better towards patients.

Some permanent staff considered the unlicensed staff a nuisance to work with, and did not expect their participation. Jacobsen [42] also found that the assistants were a “fellowship of those who have no say” (p 86). For instance, during meals [20] with many cognitively able residents the local staff’s involvement with the new residents could shut the weekend supply staff off from participation. The enthusiastic allies were good at what they were doing and the supply staff could feel redundant. That the assistants kept a distance in staff interactions, may also suggest that this legitimized their at times poor involvement with the new resident. The licensed staff may have to compensate for the assistants’ limited contributions towards the new residents, or as the findings suggest, some needs were ignored. Many auxiliaries handled the distance and difference from the nurses and themselves by the appreciation of the homelike, everyday activities, where the nurses also participated; here everybody was of equal worth. According to Gullestad [43], sameness and being of equal worth relate closely in Norwegian culture. In order to be of equal worth, one has to be the same as. Moreover, shortly after a resident had moved in, the primary auxiliaries had authority in oral interactions. The oral culture in this LTCF allowed the auxiliaries to some extent to control the information flow of the new residents’ everyday needs. If the nurses were preoccupied with other tasks, they depended on the auxiliaries’ preliminary knowledge and insights. This is to some extent in line with Alcorn [44], who in a review of the relationship between registered nurses and healthcare assistants found that “power plays materializes through this relationship as healthcare assistants are placed in powerful positions through controlling the flow of communication between registered nurses and patients” (p 11).

The nurses in our study functioned as gatekeepers as to whether they would share medical information and knowledge about the new resident with the other nursing staff. The staff nurses also had the power to decide how they wanted to involve and supervise their staff. The findings in our study suggest that the auxiliaries at times did not work to the full of their scope. This appears to be in accordance with Spilsbury & Meyer [45], who in their UK study found that the nurses had the power to control whether the health care assistant used their skills and experience to the full. Still, the dynamic interactions between all the staff suggest the strong interdependence among them. In our previous study [20], the task of writing the handwritten care plans was delegated to the primary auxiliaries to be performed shortly after the new resident had arrived. This indicate that the nurses supported the auxiliaries’ independent contributions. Alcorn and Topping [46] found that registered nurses supported the health care assistants’ development, and that patient care was enhanced through their development.

However, some auxiliaries felt excluded since they were not involved in the residents’ medical situation, and their intra-professional alliances seemed particularly important to them. One way of understanding this mechanism is that the auxiliaries found a niche for themselves within the organization, which protected them from the inherent organizational contradiction of not being involved in every aspect of the new resident, at the same time as being involved in everything [47]. Historically, the development of professions aimed at securing and protecting exclusive areas of knowledge [48], and the nurses and physician in our study acted according to this tradition. In line with some studies [9,37,49], we found that the professional cultures challenged spontaneous inter-professional collaboration, which again influenced how the new residents were treated initially. Current political trends [9,37,50] aim at developing cooperative competence among all staff categories, also the unlicensed staff. This requires close collaboration between different levels of educational institutions, and between practice institutions and educational institutions. Clark [51] found, by examining the interface between inter-professional practice and education in a Norwegian context, that there is a need to link developments in health care practice settings with those in education, “particularly in such areas as continuing professional development, may be critical to the success of inter-professional practice and inter-professional education” (p 31).

Few professional groups work in most Norwegian LTCFs and in this LTCF only nursing staff worked on an everyday basis. That the physician only interacted with the nurses also hampered cognitive diversity. In their study of two Norwegian nursing homes, Jakobsen & Granebo [52], found that there is a need for wider multidisciplinary teams to develop variations in the approaches to the older residents. The everyday extra activities provided by some enthusiasts, seemed to move the new residents towards a healthy transition. Yet, the findings show that they may overshadow the new residents’ complex needs, and well-meant activities from the staff’s point of view may have the adverse effect on some residents. Moving into LTCF is a dramatic change in a person’s life [15-17], and the person needs time to adjust to the new situation and circumstances. However, unlike in most countries, the nurses performed hands-on care. This provided diverse and complementary contributions in the spontaneous staff interactions. Moreover, the number of nurses helped maintain a clear nurse identity, and seemed to support their self-confidence in their interaction with the auxiliaries and assistants. Yet, the findings suggest that the freedom the staff nurses had to manage their units in different ways, could at times delay the work of the facility nurse.

The reading and writing of the daily reports and care plans was inefficiently performed by many. The notion that the computer program and its standards [53] from a social point of view serve as a “means for collaboration, shared meaning and far-reaching coordination among different health care professionals” (p 207) did not seem to be the view of many staff. Spontaneous oral interaction was the most useful. According to the WHO [37], inconsistent use and understanding of language may be a barrier to inter-professional collaborative practice. Ellingsen [53] argues that standardization efforts must target a level that is acceptable for those involved. Our findings suggest that the computer program did not consider the different levels of staff, and the computer care plans did not generally seem to guide the daily care of the new residents, particularly not the licensed staff who regarded the written care plans as rough guidelines only. This is in accordance with Lanham et al. [54], who argue that complex adaptive systems contain unpredictability, and that care be conceptualized as provisional plans for actions and not detailed plans to be strictly followed.

All these complex aspects of staff interactions appeared to create stress among some, regardless of formal position, although our findings suggest that the assistants, particularly the weekend supply staff, were those who most clearly appeared aloof from the rest of the staff. The focus of attention during negative stress shifts from interactions to withdrawal [49], to preserve the individual’s dignity and self-esteem. Withdrawals may contribute to less nuanced care because they mean fewer opportunities to verbalize questions and actions, and thereby less awareness of one’s own and other’s work [55].

## Conclusions

The continuous and spontaneous staff collaborations were key activities in supporting quality care in the transition period. These interactions maintained the inclusion of all staff present, staff flexibility, information flow to some extent, and cognitive diversity, and the new resident’s emerging needs appeared met. Organizational structures, staff’s formal position, and informal staff alliances were complex and sometimes appeared contradictory. Not all the staff were necessarily included, and the new residents’ needs not always noticed and dealt with. Paying attention to the playing out of power in staff interactions appears vital to secure a healthy transition process for the older residents.

### Strengths and limitations

The rich data from this small sample size study fulfil the intention of ethnographic studies to get in-depth insight into a phenomenon. This approach is valuable since no studies so far have investigated this phenomenon, and may help extract ideas and directions in future studies with larger samples and other designs. A future survey study of a representative sample of different staff employed in LTCFs could elucidate knowledge about this topic on a greater scale. Also, future studies need to link the development in health care practice settings during older residents’ transitions into LTCF with different levels of educational institutions, to explore and encourage inter-professional collaboration.

## References

[1] Mestring, mulighet og mening. Framtidas omsorgsutfordringer. (Future challenges in long-term care: coping, possibilities and meaning.) St.meld. nr. 25 (Report no. 25 to the Storting) Oslo: Helse- og omsorgsdepartementet (Ministry of Health and Care Services). 2006. (in Norwegian).

[2] Romøren TI, Kirkevold M, Brodtkorb K, Hylen-Ranhoff A (2008). Eldre, helse og hjelpebehov. Geriatrisk sykepleie – god omsorg til den gamle pasienten. (in Norwegian). Older people, their health and their need of assistance.

[3] Roksvaag K, Texmon I: Arbeidsmarkedet for helse- og sosialpersonell fram mot år 2035. Dokumentasjon av beregninger med HELSEMOD (in Norwegian). Statistics Norway, Report 14; 2012. (in Norwegian).

[4] Husebø BS, Husebø S (2005). Nursing homes as arenas of terminal care: practical aspects. J Norwegian Physicians’ Assoc (Tidsskrift for den norske legeforening).

[5] Stone R, Harahan MF (2010). Improving the long-term care workforce serving older adults. Health Aff.

[6] Harris A, McGillis L (2012). Evidence to inform staff mix decision-making: a focused literature review. Report prepared for the Canadian Nurses Association.

[7] Lookinland S, Tiedeman ME, Crosson AET (2005). Nontraditional models of care delivery. J Nurs Adm.

[8] Yeatts DE, Cready CM (2007). Consequences of empowered CAN teams in nursing home settings: a longitudinal assessment. Gerontologist.

[9] Mickan S, Hoffman SJ, Nasmith L (2010). Collaborative practice in a global health context: Common themes from developed and developing countries. J Interprof Care.

[10] Anderson RA, Toles MP, Corazzini K, McDaniel RR, Colon-Emeric C (2014). Local interaction strategies and capacity for better care in nursing homes: a multiple case study. BMC Health Serv Res.

[11] Harrington C, Choiniere J, Goldman M, Jacobsen FF, Lloyd L, McGregor M (2012). Nursing Home Staffing Standards and Staffing Levels in Six Countries. J Nurs Scholarsh.

[12] Jacobsen F, Mekki TE (2011). Health and the changing welfare state in Norway: a focus on municipal health care for elderly sick. Ageing Int.

[13] Pöyry E (2009). Bemanning i kommunal pleie og omsorg. Econ-report no. 2009–072.

[14] Tiedeman ME, Lookinland A (2004). Traditional models of care delivery: what have we learned?. J Nurs Adm.

[15] Davies S, Nolan M (2003). «Making the best of things»: relatives’ experiences of decisions about care-home entry. Ageing Soc.

[16] Eika M, Espnes GA, Söderhamn O, Hvalvik S (2014). Experiences faced by next of kin during their older family members’ transition into long-term care in a Norwegian nursing home. J Clin Nurs.

[17] Schumacher KL, Jones PS, Meleis IA, Meleis IA (2010). Helping elderly persons in transition: a framework for research and practice. Transitions theory – middle-range and situation-specific theories in nursing research and practice.

[18] Meleis AI, Sawyer LM, Im EO, Messias DKH, Schumacher K, Meleis IA (2010). Experiencing transitions: an emerging middle-range theory. Transitions theory – middle-range and situation-specific theories in nursing research and practice.

[19] Geary CR, Schumacher KL (2014). Care transitions integrating transition theory and complexity science concepts. Adv Nurs Sci.

[20] Eika M, Espnes GA, Hvalvik S (2014). Nursing staff’s actions during older residents’ transition into long-term care facility in a nursing home in rural Norway. Int J Qual Stud Health Well-being.

[21] Rubin G, Balaji RV, Barcikowski R (2009). Barriers to nurse/nursing aide communication: the search for collegiality in a southeast Ohio nursing home. J Nurs Manag.

[22] Bowers B, Nolet K (2011). Empowering direct care workers: lessons learned from the GREEN HOUSE Model. Senior Housing Care J.

[23] Hammersley M, Atkinson P (2007). Ethnography - principles in practice.

[24] O’Reilly K (2012). Ethnographic methods.

[25] Järvinen M, Mik-Meyer N (2005). Kvalitative metoder i et interaktionistisk perspektiv, interview, observationer og dokumenter.

[26] Gubrium JF, Holstein JA (1997). The New language of qualitative method.

[27] Gadamer HM (2004). Sandhed og Metode (Truth and Method) Grundtræk av en Filosofisk Hermeneutik. Oversættelse, Indledning og Noter ved Arne Jørgensen.

[28] Allen D (2004). Ethnomethodological insights into insider outsider relationships in nursing ethnographies of health care settings. Nurs Inq.

[29] Fangen K: Deltagende observasjon 2nd edition. (Participant observation) Fagbokforlaget, Bergen; 2010. (in Norwegian).

[30] Høst H (2010). Helsefagarbeiderutdanning for voksne. (The education of adult care workers). NIFU STEP, Rapport 25.

[31] Atkinson P, Coffey A, Gubrium JF, Holstein JA (2002). Revisiting the relationship between participant observation and interviewing. Handbook of interview research – context and method.

[32] Blumer H (1954). What is wrong with social theory?”. American Sociological Review..

[33] Malterud K: Kvalitative metoder I medisinsk forskning. En innføring. 3.utgave. (Qualitative methods in medical research. An introduction. 3rd edition) Universitetsforlaget, Oslo; 2011. (In Norwegian).

[34] Anderson RA, Issel LM, McDaniel RRJ (2003). Nursing homes as complex adaptive systems: Relationship between management practice and resident outcomes. Nurse Res.

[35] Anderson RA, Ammarell N, Bailey DE, Colon-Emeric C, Corazzini K, Lekan-rutledge D (2005). The power of relationship for high quality long term care. J Nurs Care Qual.

[36] Penprase B, Norris D (2005). What nurse leaders should know about complex adaptive systems theory. Nurs Leadersh Forum.

[37] Human Resources Health Observer (2013). Interprofessional Collaborative Practice in Primary Health Care: Nursing and Midwifery Perspectives. Six case studies. 13:1-24.

[38] Majerovitz D, Mollott RJ, Rudder C (2009). We’re on the same side: Improving communication between nursing home and family. Health Commun.

[39] Leykum LK, Lanham HJ, Pugh JA, Parchman M, Anderson RA, Crabtree BF (2014). Manifestations and implications of uncertainty for improving healthcare systems: an analysis of observational and interventional studies grounded in complexity science. Implement Sci.

[40] Colon-Emeric CS, Ammarell N, Bailey D, Lekan-Rutledge D, Anderson RA, Piven ML (2006). Patterns of medical and nursing staff communication in nursing homes: Implications and insights from complexity science. Qual Health Res.

[41] Bishop CE, Weinberg DB, Leutz W, Dossa A, Pfefferle SG, Zincavage RM (2008). Nursing assistants’ job commitment: effect of nursing home organizational factors and impact on resident well-being. Gerontologist.

[42] Jacobsen F (2005). Cultural discontinuity as an organizational resource: nursing in a Norwegian nursing home.

[43] Gullestad M (2006). Plausible prejudice everyday experiences and social images of nation, culture and race.

[44] Alcorn J (2010). Elements that affect the relationship between registered nurses and health care assistants: a review of the literature. Work Based Learn e-journal.

[45] Spilsbury K, Meyer J (2005). Making claim on nursing work – exploring the work of health care assistants and the implication for registered nurses’ roles. J Res Nurs.

[46] Alcorn J, Topping AE (2009). Registered nurses’ attitudes towards the role of the healthcare assistant. Nurs Stand.

[47] Hasenfeld Y, Hasenfeld (1992). The Nature of human service organizations. Human services as complex organizations.

[48] Abbott A (1988). The systems of professions.

[49] Hall P (2005). Interprofessional teamwork: professional cultures as barriers. J Interprof Care.

[50] Meld. St. 13 (Report No.13 to the Storting) Utdanning for velferd, Samspill i praksis. (Education for welfare, Interaction in practice). Oslo: Kunnskapsdepartementet (Ministry of Education and research); 2011–2012.

[51] Clark PG (2011). Examining the interface between interprofessional practice and education: Lessons learned from Norway for promoting teamwork. J Interprof Care.

[52] Jacobsen K, Granebo R (2011). Større faglig bredde som bidrag til mer aktiv omsorg for sykehjemsbeboere. Sykepleien Forskning.

[53] Ellingsen G (2004). Tightrope Walking: Standardization Meets Local Work-Practice in a Hospital. International Journal of IT- Standards and Standardization Research.

[54] Lanham HJ, Leykum LK, Taylor BS, McCannon CJ, Lindberg C, Lester RT (2013). How complexity science can inform scale-up and spread in health care: Understanding the role of self-organization in variations across local contexts. Soc Sci Med.

[55] Berger P, Luckmann T (1966). The social construction of reality – a treatise in the sociology of knowledge.

